# Expression of full-length FOXP3 exceeds other isoforms in thymus and stimulated CD4 + T cells

**DOI:** 10.1007/s10875-024-01715-8

**Published:** 2024-04-27

**Authors:** Benita Kröger, Michael Spohn, Marion Mengel, Jan-Peter Sperhake, Benjamin Ondruschka, Reiner K. Mailer

**Affiliations:** 1 Institute of Clinical Chemistry and Laboratory Medicine, University Medical Center Hamburg-Eppendorf, Hamburg, Germany; 2 Bioinformatics Core, University Medical Center Hamburg-Eppendorf, Hamburg, Germany; 3 Institute of Legal Medicine, University Medical Center Hamburg-Eppendorf, Hamburg, Germany

To the Editor

Different isoforms of the transcription factor FOXP3 orchestrate gene expression and function in human CD4 + T cells. Mutations of the *FOXP3* gene can cause fatal autoimmunity even when an alternatively spliced transcript, which is predominantly expressed in blood, is unaffected [1]. Herein, we found that FOXP3 induction initially includes preferential expression of exon 2 in the thymus and in stimulated naïve T cells in advance of a more balanced isoform ratio. Thus, the control of T-cell functions by FOXP3 may depend on timely processing of *FOXP3* transcripts.

The transcription factor FOXP3 is indispensable for the development and maintenance of anti-inflammatory CD4 + regulatory T (Treg) cells. Mutation of the *FOXP3* gene leads to failure of Treg cell-mediated tolerance against self-antigens and causes autoimmunity early in life, characterized as IPEX (immunodysregulation, polyendocrinopathy, enteropathy, X-linked syndrome) [2]. Human but not murine Treg cells express isoforms, that lack coding exon 2 (FOXP3Δ2) or exon 2 and 7 (FOXP3Δ2Δ7) in addition to full-length FOXP3 (FOXP3fl) [3, 4]. Exclusion of *FOXP3 exon 7* abrogates FOXP3fl-mediated induction of Treg-cell functions and promotes the differentiation of pro-inflammatory Th17 cells [5]. In contrast, over-expression of FOXP3Δ2 in CD4 + T cells promotes Treg-cell functions in cooperation with FOXP3fl [6]. Notably, FOXP3fl appears to be crucial for the induction of its own transcription as IPEX mutations within *FOXP3 exon 2* abrogate FOXP3 expression and Treg-cell development [3]. However, spatio-temporal expression levels of FOXP3fl and FOXP3Δ2 and their function for Treg-cell development in the thymus remained unknown so far.

To test whether alternative splicing of *FOXP3 exon 2* differs during Treg-cell development, we analyzed FOXP3 isoform expression in human thymi (median age: 4 months). We found that *FOXP3* transcripts mainly included *exon 2* and that FOXP3fl is the predominantly expressed isoform in human thymocytes detected by real-time PCR using splice-specific primers and immunoblots using antibodies (clone eBio7979) that bind to all FOXP3 isoforms, respectively (Fig. 1, A and B). Consistently, flow cytometry analysis showed that thymocytes stained with antibodies that recognize FOXP3 exon 2 (clone 150D/E4) and total FOXP3 (clone 236A/E7, that recognizes a non-spliced FOXP3 region) display fluorescence intensity (FI) ratios similar in scale to HEK-293 cells overexpressing FOXP3fl (Fig. 1C). In contrast, reduced FOXP3 FI ratios of peripheral Treg cells resemble the pattern in HEK-293 cells transfected with FOXP3Δ2 proportionally. Thus, exclusion of *FOXP3 exon 2* is diminished and enhanced FOXP3fl expression is associated with Treg-cell development in the thymus. These results are corroborated by single-cell RNAseq data [7], that found *FOXP3exon2* + */FOXP3exon2-* transcript ratios are higher among thymocytes compared to blood cells (Fig. 1D).

**Fig. 1:**
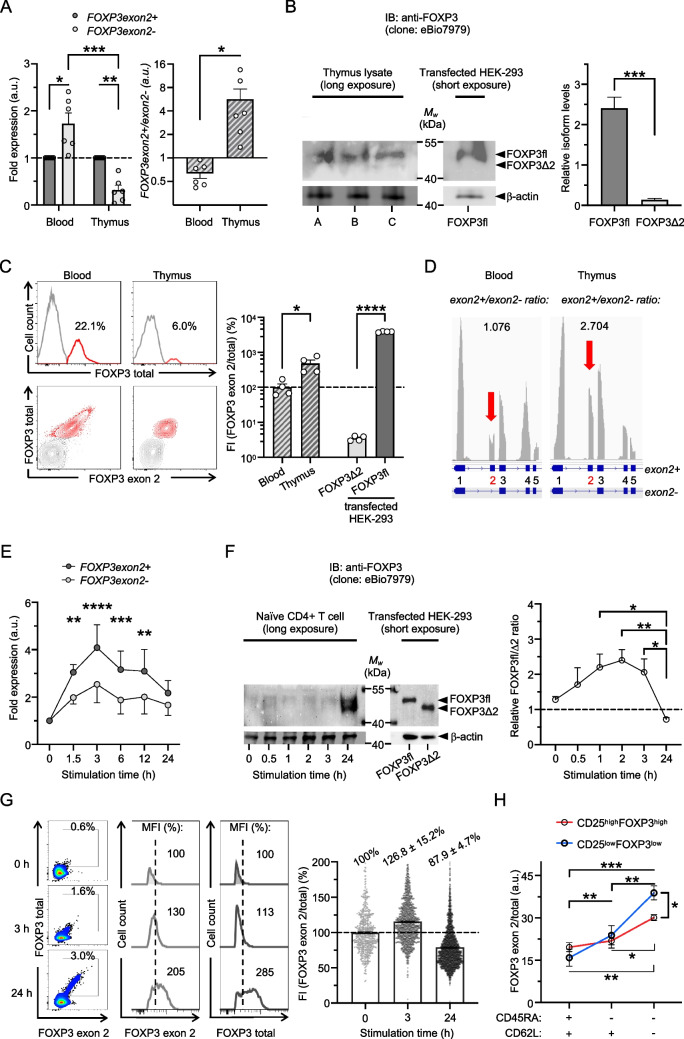
Full-length FOXP3 isoform is predominantly expressed in human thymocytes and exceeds induction of other isoforms in TCR-stimulated naïve CD4 + T cells

T-cell antigen receptor (TCR) stimulation is known to induce transient *FOXP3* transcription in naïve CD4 + T cells and increased FOXP3 exon 2 expression is associated with an activated T-cell phenotype in chronic disease [8, 9]. To investigate differential alternative splicing in response to stimulation, we analyzed FOXP3 isoform expression in human naïve CD4 + T cells stimulated with anti-CD3 antibodies (clone UCHT1). We found that *FOXP3* expression is swiftly induced and that transcripts with *FOXP3 exon 2* exceed those lacking *FOXP3 exon 2* (Fig. 1E). Moreover, initial induction of FOXP3fl was detectable by faint bands within 3 h after stimulation of naïve T cells by immunoblotting, whereas strong bands for both, FOXP3fl and FOXP3Δ2 were observed after 24 h stimulation (Fig. 1F). More expression of FOXP3fl in comparison to other isoforms was confirmed by flow cytometry analysis (Fig. 1G). FOXP3 exon 2 detection exceeded recognition of total FOXP3 in naïve T-cell populations and single-cell FI ratios increased after 3 h stimulation. Vice versa, detection levels reversed in naïve T-cell populations and single-cell FI ratios decreased after 24 h stimulation, indicating a delayed expression of FOXP3Δ2 in TCR-stimulated naïve CD4 + T cells. Consistent with an activation-induced FOXP3 isoform profile, the portion of FOXP3 exon 2 among total FOXP3 expression increased from CD4 + CD45RA + CD62L + naïve T-cell populations to CD4 + CD45RA-CD62L + and CD4 + CD45RA-CD62L- effector T-cell populations (Fig. 1H). Notably, isoform ratios increased stronger in T cells with low, compared to high, expression of CD25 and FOXP3, indicating a crucial role of the full-length isoform upon FOXP3 induction.

In conclusion, we show that initial FOXP3 induction implicates the expression of FOXP3fl during thymic Treg-cell development, whereas little FOXP3Δ2 is present at first. The predominant expression of FOXP3fl in the thymus fits to the notion that mutations of *FOXP3 exon 2* can cause IPEX [1]. Thus, our results offer an explanation why FOXP3Δ2 cannot compensate for the loss of FOXP3fl and indicate a pivotal role of FOXP3 exon 2 during the development of Treg progenitor cells in the thymus. Moreover, a preceding induction of higher FOXP3fl/FOXP3Δ2 ratios in stimulated naïve CD4 + T cells may explain impaired regulation of T-cell responses in IPEX patients with *FOXP3 exon 2* mutations, underlining isoform-specific functions and the necessity for the expression of both FOXP3fl and FOXP3Δ2 to maintain a Treg-like cell phenotype [6]. In line with these results in human T cells, unstable *Foxp3* gene expression and subsequent autoimmunity has been reported in mice with a genetic ablation of *Foxp3 exon 2* [3]. Thus, FOXP3fl appears to be important as the pioneering isoform for transcriptional control, that may suggest refined gene therapy strategies for IPEX patients to restore physiological splicing ratios of *FOXP3 exon 2*.

Human CD4 + T cells were isolated from blood of healthy donors (Miltenyi) and thymocytes from tumor-free individuals were analyzed following cell strainer filtration (100 μm, Sarstedt) of thymus samples collected in the Institute of Legal Medicine at the University Medical Center Hamburg-Eppendorf. **A)** Quantification of *FOXP3* transcripts including (*FOXP3exon2* +) or excluding (*FOXP3exon2-*) *exon 2* in human blood T cells or human thymocytes was performed by real-time PCR on StepOnePlus instrument (Applied Biosystems) using RNA extraction (RNeasy, Qiagen), cDNA generation (SuperScript IV VILO, ThermoFisher) and specific primers for *FOXP3* transcripts that include *exon 2* (Hs01092118_g1, ThermoFisher) or exclude *exon 2* (Hs03987537_m1, ThermoFisher) to quantify *FOXP3exon2* + and *FOXP3exon2-* mRNA, respectively. Relative gene expression was normalized to house-keeping gene hypoxanthine–guanine-phosphoribosyltransferase (Hs02800695_m1, ThermoFisher) (*n* = 6); fold expression in relation to *FOXP3exon2* + (left) and isoform ratios (right) are shown; unpaired t-test and one-sample Wilcoxon test for relative values was performed for statistical analysis. **B)** Immunoblotting of thymus lysates from three individuals (5 × 10^7^ thymocytes/lane A-C) probed for anti-FOXP3 (clone eBio7979, ThermoFisher) and followed by incubation with peroxidase-conjugated secondary antibody (rabbit anti-mouse, ThermoFisher). Lysate from HEK-293 cells transfected with pcDNA3.1 + plasmid encoding FOXP3fl (Lipofectamine 3000 Transfection Reagent, ThermoFisher) was used for size comparison. Chemiluminescence of substrate (ECL Select Western Blotting Detection Reagent, Amersham) was detected with ChemiDoc instrument (BioRad) and relative quantity of bands was assessed using Image Lab (Bio-Rad); paired t-test was performed for statistical analysis. **C)** Flow cytometry analysis of human CD4 + T cells and thymocytes (gating strategy depicted in Supplementary Fig. 1A) using antibodies for FOXP3 exon 2 (clone 150D/E4, ThermoFisher), which binds specifically exon 2, and FOXP3 total (clone 236A/E7, ThermoFisher), which recognizes a non-spliced region of exon 3 to exon 6, in CD4 + cells (clone: RPA-T4, BioLegend). A single-cell parameter [(FOXP3 exon2 fluorescence intensity)/(FOXP3 total fluorescence intensity)] was derived to compare FOXP3 isoform ratios in samples [9]. The average of single-cell fluorescence intensity (FI) ratios in relation to blood samples is shown (*n* = 4); FOXP3fl and FOXP3Δ2 transfected HEK-293 cells (Supplementary Fig. 1B) are shown for comparison; unpaired t-test was performed for statistical analysis. **D)** FOXP3 exon 2 transcript usage map (pile-up track, gray) with calculated *exon2* + */exon2-* ratios of differential abundances of *FOXP3exon2* + *and FOXP3exon2- mRNA* in transcript per million and genomic coordinates (blue) from publicly available RNAseq data files for human PBMCs (www.10xgenomics.com/datasets, whole transcriptome analysis v1.1, Cell Ranger 4.0.0) and human thymocytes (GSE148978_RAW), respectively. All samples were analyzed as pseudo-bulk experiments as identification of transcript per million of single cells was prevented by limited reads per cell. Naïve CD4 + T cells from healthy donors were isolated (Naïve CD4 + T Cell Isolation Kit, Miltenyi) and induction of FOXP3 isoforms following TCR stimulation with 3 μg/ml plate-bound anti-CD3 antibody (clone UCHT1, Invitrogen) in serum-free media (X-VIVO 15, Lonza) was analyzed at indicated time points by **E)** real-time PCR and two-way ANOVA followed by Sidak’s multiple comparisons test (*n* = 5), **F)** immunoblotting (1 × 10^6^ cells/lane) and densitometry with one-way ANOVA followed by Tukey’s multiple comparisons test (*n* = 4) and **G)** representative flow cytometry analyses as described above, values indicate population size and mean fluorescence intensities (MFI) of samples (left) and population averages of FI ratios (right) (*n* = 3). **H)** Mean values of single-cell parameter [FI(FOXP3 exon2)/FI(FOXP3 total)] for peripheral CD4 + T-cell populations with differential expression of CD45RA (clone: UCHL1, BioLegend), CD62L (clone: DREG-56, BioLegend) and CD25 (clone: M-A251, BioLegend) is shown; one-way ANOVA followed by Tukey’s multiple comparisons test was performed for statistical analysis (*n* = 3). Values are expressed as mean ± SEM; *P < 0.05, **P < 0.01, ***P < 0.001, ****P < 0.0001.

### Supplementary Information

Below is the link to the electronic supplementary material.

Supplementary file1 (PDF 134 KB)

## Data Availability

The datasets generated during and/or analysed during the current study are available from the corresponding author on reasonable request.

## References

[1] Mailer RK (2020). IPEX as a Consequence of Alternatively Spliced FOXP3. Front Pediatr.

[2] Wildin RS, Ramsdell F, Peake J, Faravelli F, Casanova JL, Buist N, Levy-Lahad E, Mazzella M, Goulet O, Perroni L, Bricarelli FD, Byrne G, McEuen M, Proll S, Appleby M, Brunkow ME (2001). X-linked neonatal diabetes mellitus, enteropathy and endocrinopathy syndrome is the human equivalent of mouse scurfy. Nat Genet.

[3] Du J, Wang Q, Yang S, Chen S, Fu Y, Spath S, Domeier P, Hagin D, Anover-Sombke S, Haouili M, Liu S, Wan J, Han L, Liu J, Yang L, Sangani N, Li Y, Lu X, Janga SC, Kaplan MH, Torgerson TR, Ziegler SF, Zhou B (2022). FOXP3 exon 2 controls T(reg) stability and autoimmunity. Sci Immunol.

[4] Mailer RK, Falk K, Rotzschke O (2009). Absence of leucine zipper in the natural FOXP3Delta2Delta7 isoform does not affect dimerization but abrogates suppressive capacity. PLoS ONE.

[5] Mailer RK, Joly AL, Liu S, Elias S, Tegner J, Andersson J (2015). IL-1beta promotes Th17 differentiation by inducing alternative splicing of FOXP3. Sci Rep.

[6] Sato Y, Liu J, Lee E, Perriman R, Roncarolo MG, Bacchetta R (2021). Co-Expression of FOXP3FL and FOXP3Delta2 Isoforms Is Required for Optimal Treg-Like Cell Phenotypes and Suppressive Function. Front Immunol.

[7] Chopp LB, Gopalan V, Ciucci T, Ruchinskas A, Rae Z, Lagarde M, Gao Y, Li C, Bosticardo M, Pala F, Livak F, Kelly MC, Hannenhalli S, Bosselut R (2020). An Integrated Epigenomic and Transcriptomic Map of Mouse and Human alphabeta T Cell Development. Immunity.

[8] Walker MR, Kasprowicz DJ, Gersuk VH, Benard A, Van Landeghen M, Buckner JH, Ziegler SF (2003). Induction of FoxP3 and acquisition of T regulatory activity by stimulated human CD4+CD25- T cells. J Clin Invest.

[9] Lundberg AK, Jonasson L, Hansson GK, Mailer RKW (2017). Activation-induced FOXP3 isoform profile in peripheral CD4+ T cells is associated with coronary artery disease. Atherosclerosis.

