# Supplementary material for: Expression of full-length FOXP3 exceeds other isoforms in thymus and stimulated CD4 + T cells
Source: J Clin Immunol. 2024 Apr 27;44(5):114. doi: 10.1007/s10875-024-01715-8 (PMC11055749; doi:10.1007/s10875-024-01715-8)
Supplement: Supplementary file 1 — Supplementary file1 (PDF 134 KB) [file 10875_2024_1715_MOESM1_ESM.pdf]

A

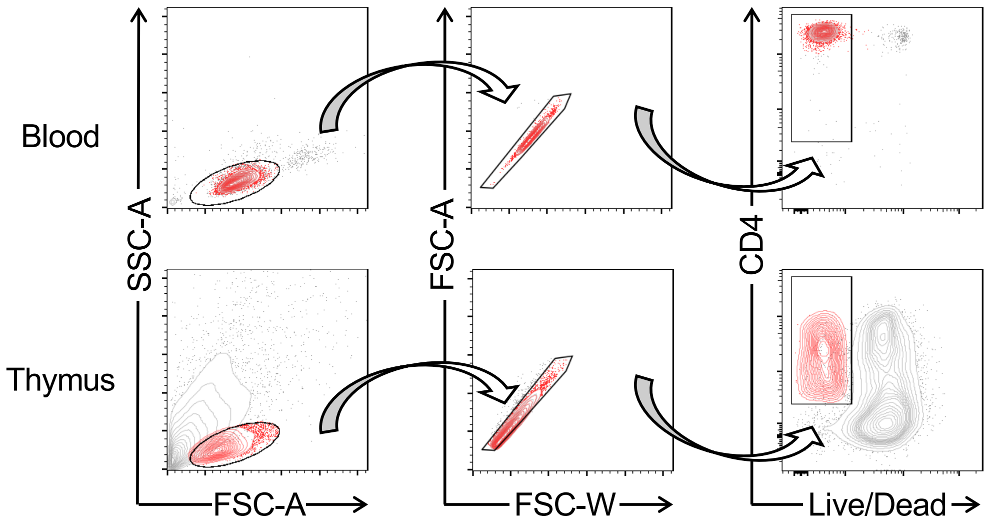

Flow cytometry analysis gating strategy. FACS analysis of peripheral CD4+ T cells and thymocytes was performed on cells within the lymphocyte gate of forward/side scatter plots, excluding doublets and dead cells (LIVE/DEAD Fixable Aqua Dead Cell Stain Kit, Invitrogen). Events were acquired on FACSCantoII cytometer (BD Biosciences) and data were analyzed with FlowJo v10.6.1 software (Treestar).

B

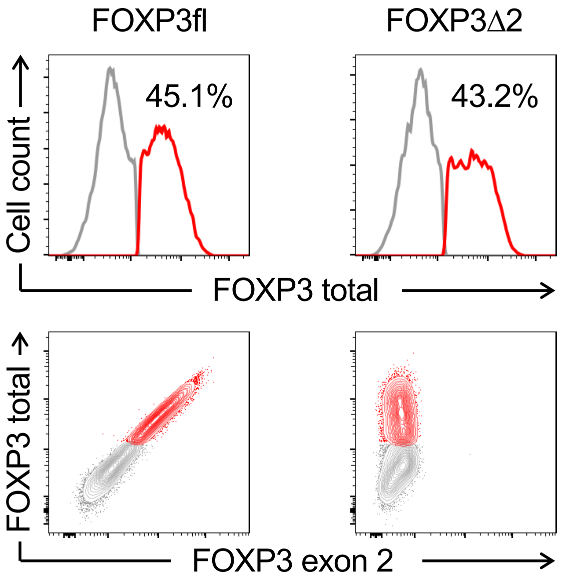

Exogenous FOXP3 isoform detection in HEK-293 cells. FACS analysis of HEK-293 cells transfected with FOXP3fl or FOXP3Δ2 using antibodies for FOXP3 total (clone 236A/E7, which recognizes a non-spliced region of exon 3 to exon 6) and FOXP3 exon 2 (clone 150D/E4, which binds specifically exon 2), as described previously (Lord et al., *Dig Dis Sci* 2012; De Rosa et al., *Nat Immunol* 2015; Mailer, *Front Immunol* 2018).
